# Extreme heat increases stomatal conductance and drought‐induced mortality risk in vulnerable plant species

**DOI:** 10.1111/gcb.15976

**Published:** 2021-11-20

**Authors:** Renée M. Marchin, Diana Backes, Alessandro Ossola, Michelle R. Leishman, Mark G. Tjoelker, David S. Ellsworth

**Affiliations:** ^1^ Hawkesbury Institute for the Environment Western Sydney University Penrith New South Wales Australia; ^2^ Department of Biological Sciences Macquarie University North Ryde New South Wales Australia

**Keywords:** drought stress, heatwave, high temperature tolerance, leaf critical temperature, thermal safety margin, water deficit experiment

## Abstract

Tree mortality during global‐change‐type drought is usually attributed to xylem dysfunction, but as climate change increases the frequency of extreme heat events, it is necessary to better understand the interactive role of heat stress. We hypothesized that some drought‐stressed plants paradoxically open stomata in heatwaves to prevent leaves from critically overheating. We experimentally imposed heat (>40°C) and drought stress onto 20 broadleaf evergreen tree/shrub species in a glasshouse study. Most well‐watered plants avoided lethal overheating, but drought exacerbated thermal damage during heatwaves. Thermal safety margins (TSM) quantifying the difference between leaf surface temperature and leaf critical temperature, where photosynthesis is disrupted, identified species vulnerability to heatwaves. Several mechanisms contributed to high heat tolerance and avoidance of damaging leaf temperatures—small leaf size, low leaf osmotic potential, high leaf mass per area (i.e., thick, dense leaves), high transpirational capacity, and access to water. Water‐stressed plants had smaller TSM, greater crown dieback, and a fundamentally different stomatal heatwave response relative to well‐watered plants. On average, well‐watered plants closed stomata and decreased stomatal conductance (*g*_s_) during the heatwave, but droughted plants did not. Plant species with low *g*_s_, either due to isohydric stomatal behavior under water deficit or inherently low transpirational capacity, opened stomata and increased *g*_s_ under high temperatures. The current paradigm maintains that stomata close before hydraulic thresholds are surpassed, but our results suggest that isohydric species may dramatically increase *g*_s_ (over sixfold increases) even past their leaf turgor loss point. By actively increasing water loss at high temperatures, plants can be driven toward mortality thresholds more rapidly than has been previously recognized. The inclusion of TSM and responses to heat stress could improve our ability to predict the vulnerability of different tree species to future droughts.

## Graphical abstract

Tree death during drought occurs when critical water transport from soil to plant leaves is interrupted, causing catastrophic desiccation, but the key role of extreme heat in this process remains unclear. We found evidence that multiple plant species actively increased water loss during heatwaves, a process that simultaneously cools leaves but sacrifices water, driving droughted plants rapidly toward mortality thresholds. This response is not yet captured in plant water use models and may help identify which species are vulnerable to hotter drought events.

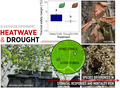

## 1 INTRODUCTION

Forests around the globe are under threat, with many showing signs of decline and changes in community composition as a result of climate stress (Allen et al., 2010; Brodribb et al., 2020; Trugman et al., 2020). The term “global‐change‐type drought” is used to describe the warmer, longer, and more frequent droughts (Breshears et al., 2005) that are now plaguing many forests worldwide. Drought stress is usually considered to be the driving factor for mortality during large‐scale forest dieback events (Choat et al., 2018), in part because hydraulic water‐transport traits have been associated with global patterns of forest mortality (Anderegg et al., 2016). The role of chronic warming in driving tree mortality is usually described as indirect—high temperatures and the associated increase in atmospheric vapor pressure deficit (VPD) and evapotranspiration results in enhanced soil drying and increased drought severity (Allen et al., 2015; Williams et al., 2013), ultimately causing hydraulic failure of the plant water transport system (Adams et al., 2017). The most widely recognized mechanisms causing tree death during drought are xylem cavitation, carbon starvation, and biotic interactions with insects and/or pathogens (McDowell et al., 2011). Much less attention has been given to the direct effect of high temperature as a possible mechanism of tree death during drought (Breshears et al., 2021), despite the fact that acute heatwaves often coincide with global‐change‐type droughts (Teskey et al., 2015). Many trees will be increasingly exposed to extreme drought (Xu et al., 2019) and heatwaves (Perkins‐Kirkpatrick & Lewis, 2020) in the future, with recent heatwaves spanning massive areas that cross multiple ecosystems (Ruthrof et al., 2018).

Plants can endure very high air temperatures (*T*_air_) by dissipating heat through conduction, convection, and evaporative cooling, allowing tolerance of some of the highest air temperatures on Earth (Hüve et al., 2011). Plants under severe drought have partially or fully closed stomata, however, which limits evaporative cooling via transpiration (Urban et al., 2017) and pushes plants closer to critical temperature thresholds. Cell death can occur within minutes of exposure to high leaf temperature (*T*_leaf_) (Hüve et al., 2011), and considerable energy is required for physiological toleration of plant heat stress (Wahid et al., 2007). Plant species differ in their vulnerability to heatwaves (Lancaster & Humphreys, 2020; O'Sullivan et al., 2017), which can be assessed using leaf thermal safety margins (TSM)—the difference between species’ photosynthetic temperature tolerance (i.e., leaf critical temperature for chloroplast survival, *T*_crit_) and *T*_leaf_. The *T*_crit_ is the high temperature where chlorophyll *a* fluorescence rises rapidly (Schreiber & Berry, 1977), indicating disruption of photosystem II (PSII) and the onset of irreversible tissue damage (Bilger et al., 1984). Relatively few studies have quantified TSM using *T*_leaf_ to date (but see Cook et al., 2021; Perez & Feeley, 2020), perhaps because *T*_leaf_ changes dynamically as a function of varying environmental conditions (solar radiation, *T*_air_, vapor pressure, wind speed) plus sensible and latent heat fluxes (Gutschick, 2016). This integrative measure combines multiple aspects of plant physiology and leaf energy balance, namely *T*_crit_ and *T*_leaf_, and holds promise for understanding which species are most vulnerable to high *T*_air_.

Plants rely on transpiration and evaporative cooling to prevent thermal damage (Schymanski et al., 2013), but little is known about the direct effect of temperature on stomata and stomatal conductance (*g*_s_). Previous experiments have revealed a range of responses, from stomatal opening (Aparecido et al., 2020; Drake et al., 2018; Marchin et al., 2016; Urban et al., 2017) to stomatal closure with increasing temperature (Hamerlynck & Knapp, 1996; Lahr et al., 2015; Mott & Peak, 2010; Slot et al., 2016), or a lack of significant temperature response. One complication is that the effect of temperature on stomatal aperture is confounded by concurrent changes in VPD with warming (Amthor et al., 2010); plant stomata are highly sensitive to VPD, which has climbed exponentially due to increasing *T*_air_ in recent decades (Grossiord et al., 2020). Undoubtedly, intra‐ and interspecies differences in the temperature sensitivity of stomata also exist and contribute to inconsistencies in observed responses of stomata to high temperature. The dynamic behavior of stomata in regulating water loss dictates the rate of plant dehydration as soil water availability declines, so understanding the high‐temperature response of stomata is essential for predicting tree death from hotter droughts.

Although forecasting tree mortality under drought is not yet possible (Choat et al., 2018), merging stomatal responses with whole‐plant water use provides a promising framework for predicting species’ vulnerability to drought (Skelton et al., 2015) and perhaps also to heatwaves. Plant stomatal behavior can be described along a continuum from isohydric to anisohydric (Klein, 2014), corresponding to plant drought strategies that range from avoidance to tolerance. Anisohydric behavior is defined by large declines in leaf water potential (Ψ_leaf_) and greater dehydration during drought, whereas isohydric behavior is associated with stomatal closure to prevent declines in Ψ_leaf_ and avoid dehydration (Tardieu & Simonneau, 1998). Much research has contributed to identifying plant functional traits associated with the isohydric‒anisohydric framework, leading to an emerging understanding of how stomatal responses are coordinated with trade‐offs among water transport traits. Anisohydric species generally have higher drought tolerance due to embolism‐resistant xylem and thick, dense leaves with lower turgor loss points (Fu & Meinzer, 2019; Zhu, Chen, et al., 2018). Isohydric species have been shown to maintain larger hydraulic safety margins (Skelton et al., 2015; Zhu, Chen, et al., 2018) and use drought‐avoidance strategies, such as drought deciduousness or deep rooting systems, to prevent hydraulic failure and leaf desiccation during dry periods (Hoffmann et al., 2011). Yet, it remains unclear whether isohydric or anisohydric species are generally more vulnerable to drought (Fu & Meinzer, 2019) or how stomata of isohydric or anisohydric species will respond to heatwaves. Given differences in water‐relations behavior, it is reasonable to expect that stomatal responses at heat extremes could differ between isohydric and anisohydric species. Clarifying this could help predict species vulnerability and enable management interventions that minimize the widespread tree mortality resulting from climate changes.

Tree death is triggered when critical hydraulic or thermal thresholds are surpassed (Breshears et al., 2021; Choat et al., 2018), and here we focus on the direct effect of heat stress on plant mortality. We experimentally imposed the combination of heat and drought stress onto 20 broadleaf evergreen tree/shrub species in glasshouse experiments. The selected species naturally occur in a wide and diverse range of Australasian environments with mean maximum monthly temperatures ranging from 25 to 36°C (Table S1), spanning low to high dehydration tolerance (Table 1), allowing us to examine fundamental hypotheses about plant heat and drought tolerance. Soil water content was gradually decreased for half of the potted plants over a period of 5 weeks to simulate a realistic drought with moderate intensity (Marchin et al., 2020), before all plants were exposed to a 6‐day heatwave with a maximum air temperature of 42°C (Figure S1). We measured leaf TSM to assess species’ vulnerability to heatwaves and compared stomatal responses across species to better understand how stomata respond to heat. We hypothesized that: (1) TSM are closely related to thresholds for leaf death and crown dieback during experimental heatwaves, with drought‐stressed plants having smaller TSM and greater damage relative to well‐watered plants, (2) drought‐stressed plants paradoxically open stomata and increase *g*_s_ to prevent leaves from critically overheating during heatwaves, and (3) species with anisohydric stomatal behavior maintain higher *g*_s_ relative to isohydric species under combined heat and drought stress. An improved understanding of how trees respond physiologically to the combination of heatwaves and drought is central to predicting the effect of extreme climate events on terrestrial ecosystems.

**TABLE 1:** Twenty broadleaf evergreen study species were ranked along the isohydric to anisohydric continuum by integrating data from leaf water potential at turgor loss point (*π*_tlp_), wood density, and two physiological responses to drought: the decrease in mean midday leaf water potential (Ψ_leaf_) and mean relative stomatal conductance (1 − *g*_s,drought_/*g*_s,control_) of droughted plants, relative to well‐watered, control plants. Values are means (±*SE*) of 4–9 plants per treatment

Species	Relative iso/anisohydry ranking	*π*_tlp_ (MPa)	Wood density (g cm^−3^)	Drought ΔΨ_leaf_ (MPa)	Relative drought *g*_s_ (%)
*Banksia serrata*	Isohydric	‒1.61 ± 0.03	0.38 ± 0.01	0	92
*Banksia robur*	Isohydric	‒1.87 ± 0.04	0.46 ± 0.02	−0.08	98
*Ficus microcarpa*	Isohydric	‒1.75 ± 0.02	0.47 ± 0.02	−0.57	97
*Callistemon citrinus*	Isohydric	‒1.40 ± 0.02	0.53 ± 0.02	−0.81	94
*Flindersia brayleyana*	Isohydric	‒1.90 ± 0.03	0.47 ± 0.02	−0.46	86
*Cupaniopsis anacardioides*		‒1.86 ± 0.03	0.47 ± 0.01	−0.52	75
*Stenocarpus sinuatus*		‒2.13 ± 0.03	0.51 ± 0.03	−0.42	80
*Atractocarpus fitzalanii*		‒2.08 ± 0.02	0.55 ± 0.02	−0.80	91
*Xanthostemon chrysanthus*		‒1.79 ± 0.04	0.59 ± 0.01	−0.88	69
*Eremophila bignoniiflora*		‒1.64 ± 0.02	0.65 ± 0.02	−1.37	91
*Backhousia citriodora*		‒1.99 ± 0.03	0.61 ± 0.01	−0.28	84
*Alectryon coriaceus*		‒1.89 ± 0.03	0.64 ± 0.02	−0.60	81
*Syzygium wilsonii*		‒1.98 ± 0.04	0.49 ± 0.01	−1.69	73
*Eucalyptus populnea*		‒2.01 ± 0.03	0.59 ± 0.01	−1.40	93
*Backhousia myrtifolia*		‒1.96 ± 0.04	0.64 ± 0.01	−0.78	75
*Dysoxylum fraserianum*	Anisohydric	‒2.16 ± 0.07	0.57 ± 0.02	−1.00	87
*Alectryon oleifolius*	Anisohydric	‒2.47 ± 0.03	0.59 ± 0.01	−1.27	60
*Flindersia xanthoxyla*	Anisohydric	‒2.45 ± 0.05	0.61 ± 0.03	−0.81	59
*Flindersia australis*	Anisohydric	‒2.14 ± 0.03	0.69 ± 0.03	−1.04	60
*Murraya paniculata*	Anisohydric	‒3.07 ± 0.15	0.72 ± 0.02	−3.44	84

## 2 MATERIALS AND METHODS

### 2.1 Study species and glasshouse experiments

Twenty broadleaf evergreen tree/shrub species were selected from a wide range of habitats throughout Australasia, from tropical rainforests to semi‐arid woodlands (Table S1). Planting stock (*n *= 10 plants per species) ranged from tubestock to 140‐ and 200‐mm pot size and was obtained from commercial nurseries in Australia located near the centroid of the species’ range, whenever possible. For five species with large ranges, an additional provenance (*n* = 5‒10 plants per species) was obtained from another nursery to provide a better representation of species’ traits. Seedlings were bare‐rooted and transplanted into 6‐L square pots containing native potting mix (<30% sand/coir, >70% screened composted pine bark; Australian Growing Solutions), 38 g of controlled‐release native plant fertilizer (Scotts Australia Osmocote Slow Release), and 1.25 g of systemic insecticide and fertilizer tablet (Yates Confidor).

Plants were grown in one of two coordinated glasshouse experiments at the Hawkesbury Institute for the Environment (Western Sydney University) from November 1, 2017 to March 23, 2018 (Experiment 1) or October 1, 2018 to February 8, 2019 (Experiment 2). Three species (*Atractocarpus fitzalanii*, *Dysoxylum fraserianum*, *Syzygium wilsonii*) were grown in both experiments; results from all replicates were pooled. Seedlings were randomly rotated within and between glasshouse bays on a monthly basis to allow uniform solar irradiance for growth. All seedlings were well‐watered using drip irrigation for 6–15 weeks to establish roots, allow the formation of new leaves, and acclimate to the glasshouse environment. Watering to saturation required 1 L at 6:00 at the beginning of the experiment and was increased to a maximum of 4.5 L daily (delivered at 8:00, 13:00, and 17:00) as plants grew larger.

The average glasshouse temperature was 28°C with a diurnal range from 22 to 35°C (Figure S1b) to represent summer conditions in southeastern Australia. Species in Experiment 1 were unintentionally exposed to high temperatures (>42°C) at midday due to glasshouse cooling malfunctions within the month preceding their measurement dates. As a result, mean leaf critical temperature (*T*_crit_) was 3.4°C higher in Experiment 1, relative to Experiment 2 (*F*_1,190_ = 81.28, *p* < .001), but there was no significant difference in the mean crown dieback between the experiments (*F*_1,209_ = 0.34, *p* = .563). Daily maximum photosynthetically active radiation (PAR) was >2000 µmol m^−2^ s^−1^, daytime relative humidity was 40%–95%, and daytime VPD was 0.2–3.8 kPa inside the glasshouse (Figure S1).

After the acclimation period, half of the plants (*n* = 5 plants per species) were exposed to a gradual, 5‐week drought (Figure S2a) following the method described in Marchin et al. (2020). Soil volumetric water content (VWC) was monitored weekly using a 20‐cm soil water content probe (CS658 HydroSenseII; Campbell Scientific Inc.) for the first 3 weeks of the experimental drought treatment, but every 3 days thereafter during the plant measurement weeks; soil VWC was always measured in the morning (8:00–10:00). The target drought intensity was 7.5 ± 2.5% soil VWC, which was below the permanent wilting point of the soil (14%).

In the final week of water deficit (days 29–35), all plants were exposed to a 6‐day heatwave that was +7°C above baseline temperatures (average daily temperature: 35°C, maximum midday temperature: 42°C; Figure S1b). Watering regimes of control and droughted plants were continued during the experimental heatwave—control pots were well‐watered using drip irrigation, while drought pots were maintained on foam with 22‐cm depth to the water table. On measurement days, soil VWC was 2%–13% for drought pots and 17%–45% for control pots (Figure S2b). It was not possible to measure all species in the same week due to logistic constraints, so 2–3 species were batched and treated at the same time.

### 2.2 Physiological measurements of plant temperature tolerance

Leaf critical temperature (*T*_crit_) was measured on control and drought plants (*n* = 3–9 plants per treatment) under baseline and heatwave conditions. The temperature‐dependent rise of steady‐state chlorophyll *a* fluorescence (*T*_leaf_ − *F*_o_) was measured in vivo on dark‐adapted leaves following the method of Schreiber and Berry (1977), with several modifications. First, plants were temporarily relocated into the laboratory, and fully‐expanded leaves were dark‐adapted for at least 10 min or until a stable fluorescence signal was achieved. Second, attached leaves were flattened and pressed firmly onto a filter paper on top of a Peltier thermoelectric cooler (Model APH‐161‐12‐18‐E; European Thermodynamics) inside a custom‐built leaf cuvette. A Peltier temperature controller (Arduino Nano V3) regulated temperature increases via a PID control algorithm and was connected to a touch‐screen computer interface (Model 3B, Raspberry Pi). One thermistor (Model MC65F103A; GE Sensing/Thermometrics) monitored temperature at the surface of the Peltier heater, while another thermistor recorded adaxial leaf temperature (*T*_leaf_); these two temperatures were averaged to estimate *T*_leaf_. Leaves were exposed to low‐intensity, far‐red illumination (<1 µmol m^−2^ s^−1^) to maintain PSII in an oxidized state (3, 4), and the end of the fiber optic cable was placed at a 60° angle to the leaf surface. The *F*_o_ was recorded every 1 s by a fluorometer (MINI‐PAM‐II/B; Heinz Walz GmbH) as Peltier temperature in the cuvette increased from 35 to 70°C at a rate of 1°C min^−1^. The *T*_crit_ was calculated as the intersection of linear slow‐ and fast‐rise phases of *T*_leaf_ − *F*_o_ curves. For the slow‐rise phase, minimum *F*_o_ was averaged from 35 to 38°C using a zero‐slope line. The fast‐rise phase was calculated using *F*_o_ from ±1.5 min of the midpoint between minimum and maximum *F*_o_ values.

Maximum *T*_leaf_ was measured on sunny days at midday (12:00–14:00) on three fully‐expanded, unshaded leaves per plant using an infrared thermometer (Agri‐Therm III Model 6110L; Everest Interscience, Inc.) held ~10 cm from the leaf surface. Control and droughted plants (*n* = 3–9 plants per treatment) were measured during baseline and heatwave conditions with thermal emissivity set to 0.92, a representative value for individual plant leaves (Jones, 2004). For a subset of five species in Experiment 1 and four species in Experiment 2, *T*_leaf_ measurements were independently validated using fine‐wire thermocouples (36‐gauge Type T; Omega) attached to the abaxial leaf surface with surgical tape; measurements were recorded every 1 min by an automated datalogger (CR1000; Campbell Scientific Inc.). Point and continuous *T*_leaf_ measurements were comparable (Figure S3), so were pooled for each plant. The TSM were calculated under heatwave conditions using the equation: $\text{TSM}=T_{\text{crit}}‐T_{\text{leaf}}$, such that negative TSM indicate *T*_leaf_ has exceeded the threshold for photosynthetic damage. Two leaf physical traits potentially related to TSM were collected: leaf size and leaf mass per area (LMA). Leaf size was measured for three fully‐expanded leaves or leaflets (for compound‐leaved species) using a flatbed scanner and the program WinRHIZO^™^ (Regent Instruments Inc.). Leaves were then oven‐dried for 48 h at 70°C to obtain dry mass.

Thermal damage after the heatwave was assessed by visually determining whole‐plant crown dieback. Two expert observers provided independent estimates of the percentage of leaf scorch/necrosis, relative to total leaf area, under baseline conditions (*D*_BS1_, *D*_BS2_), immediately after the heatwave (*D*_HW1_, *D*_HW2_), and after 2 weeks of recovery (*D*_Rec1_, *D*_Rec2_) under well‐watered conditions and baseline temperatures. Crown dieback was calculated as $\left(D_{\text{max}1}+D_{\text{max}2}/2\right)‐\left(D_{\text{BS}1}+D_{\text{BS}2}/2\right)$ for each plant, where data from both observers was averaged and maximum dieback (*D*_max1_, *D*_max2_; either after the heatwave or after recovery) was used to account for longer‐term heatwave effects. Mortality was defined as 100% crown dieback and a failure to resprout during recovery. The 2‐week recovery period was sufficient to capture subsequent mortality in all but two study plants, which had notable decline during recovery (+15%‒20% damage) and may have died at a later date.

### 2.3 Physiological measurements of plant drought responses

Species were ranked along the iso/anisohydric continuum by integrating data from species’ mean: (1) leaf water potential at turgor loss point (*π*_tlp_), (2) wood density, and (3) averaged change in midday Ψ_leaf_ and *g*_s_ under experimental drought (Table 1). The osmotic potential of fully‐expanded, fully‐hydrated leaves (*π*_o_; *n* = 4–9 plants per treatment) was measured using an osmometer (WP4C Dewpoint PotentiaMeter; Decagon Devices) according to the method described by Bartlett et al. (2012). Briefly, leaves were collected and rehydrated overnight for 12 h using the standing rehydration method (Arndt et al., 2015) to ensure fully‐hydrated leaves (i.e. Ψ_leaf_ ≥ –0.3 MPa) were used for comparison across species. The midrib was removed, leaves were frozen in LN_2_, equilibrated for 10 min, and punctured with sharp‐tipped forceps before measurement. Measurements were recorded for 20–30 min until equilibrium was reached as indicated by <0.01 MPa change over 2 min. Osmometer measurements of *π*_o_ were used to estimate species’ *π*_tlp_ using the equation from Bartlett et al. (2012): $\pi_{\text{tlp}}=0.832\pi_{o}‐0.631$. Wood density was determined for five plants per species by splitting a 5‐cm stem segment to remove the pith and bark. Fresh sapwood volume was determined using the water displacement method. Wood samples were then oven‐dried to constant mass at 105°C.

Stomatal conductance was measured at midday (10:00–14:00) on two or three fully‐expanded leaves per plant using either a porometer (AP‐4; Delta‐T) or a portable infrared gas analyzer equipped with a red–blue light source (LI‐6400XT; LI‐COR Biosciences). Control and droughted plants (*n* = 3–9 plants per treatment) were measured under baseline and heatwave conditions using a porometer in Experiment 1. The porometer failed to function under the high‐humidity heatwave conditions, so plants were individually moved into a baseline‐temperature room for 3 min to measure heatwave *g*_s_. In Experiment 2, *g*_s_ was measured using the LI‐6400XT infrared gas analyzer with leaves under saturating light (PAR = 1800 µmol m^−2^ s^−1^), ambient leaf temperature, the CO_2_ concentration of 420 ppm, and relative humidity ±10% of ambient. Instruments were calibrated either once per day (for infrared gas analyzer) or whenever ambient temperature or relative humidity shifted (by >5°C or >10%, respectively; for porometer), as appropriate for each instrument. Measurements were recorded every 5 s for 30 s after conditions inside the cuvette stabilized (usually 1–2 min) and then averaged for each leaf. The same leaves were used to measure midday Ψ_leaf_ with a pressure chamber (Model 1505D; PMS Instruments). Leaves were stored inside a sealed, humidified plastic bag and kept cool and dark until measurement (within 3 h of collection). To determine how the heat affected *g*_s_, we calculated the relative heatwave Δ*g*_s_ for each plant as $\left(g_{\text{s,heatwave}}‐g_{\text{s,baseline}}\right)/g_{\text{s,baseline}}$.

### 2.4 Statistical analyses

The effects of heat and drought on *T*_leaf_, *T*_crit_, TSM, and *g*_s_ were determined using full‐factorial, mixed‐model analyses of variance (ANOVAs) with species and treatment as the main effects; species identity was included as a random effect and treatment as a fixed effect. Additional random effects were included in models for (1) experiment, to account for the glasshouse cooling malfunctions during the first experiment, and (2) plant, to account for differences among individuals; these random effects were not included when inclusion resulted in failed model convergence or overfitting of models. Separate models were used to test for species × treatment interactions, and if significant (*p* ≤ .05), individual species’ responses were analyzed using Student's *t*‐tests. We used analyses of covariance to test for a drought effect on the *T*_crit_‒TSM, *T*_crit_‒crown dieback, leaf size‒TSM, and *π*_o_‒TSM relationships. We used linear and nonlinear regression to examine relationships between leaf size, LMA, *π*_o_, *g*_s_, *T*_leaf_, *T*_crit_, TSM, and crown dieback; the best model was selected using Akaike's information criterion, corrected for small sizes. Differences in *g*_s_ and crown dieback between isohydric and anisohydric functional groups were tested using one‐way ANOVAs with iso/anisohydry as the main effect. For isohydric and anisohydric subsets, the effect of heat on species’ mean *g*_s_ was analyzed using one‐way ANOVAs with treatment as the main effect. All data were tested for normality with the Shapiro and Wilk's test; *T*_leaf_, *T*_crit_, Ψ_leaf_, and *g*_s_ measurements were ln‐transformed to achieve normality. All statistical analyses were completed using r Statistical Software, version 3.5.1 (R Core Team, 2018). Means were considered significantly different at *p* ≤ .05; errors were expressed as standard errors of the mean (*SE*).

## 3 RESULTS

We exposed 20 broadleaf evergreen species (Table 1) to a gradual, moderate experimental drought in glasshouse experiments. Droughted plants had lower midday Ψ_leaf_ (*F*_1,190_ = 35.559, *p* < .001) than control plants by the fourth week of drought (Figure S2). All species were then exposed to an experimental heatwave with a 7°C‐increase in midday air temperature (to >41°C sustained for 6 h each day), resulting in modest crown dieback (species means: <50%) followed by resumed growth during the 2‐week recovery period. For some individual plants, however, extensive crown dieback (>50%) was observed, with plant mortality occurring for two species (*Banksia robur*: 2 plants died, *Callistemon citrinus*: 1 plant died).

**FIGURE 1:**
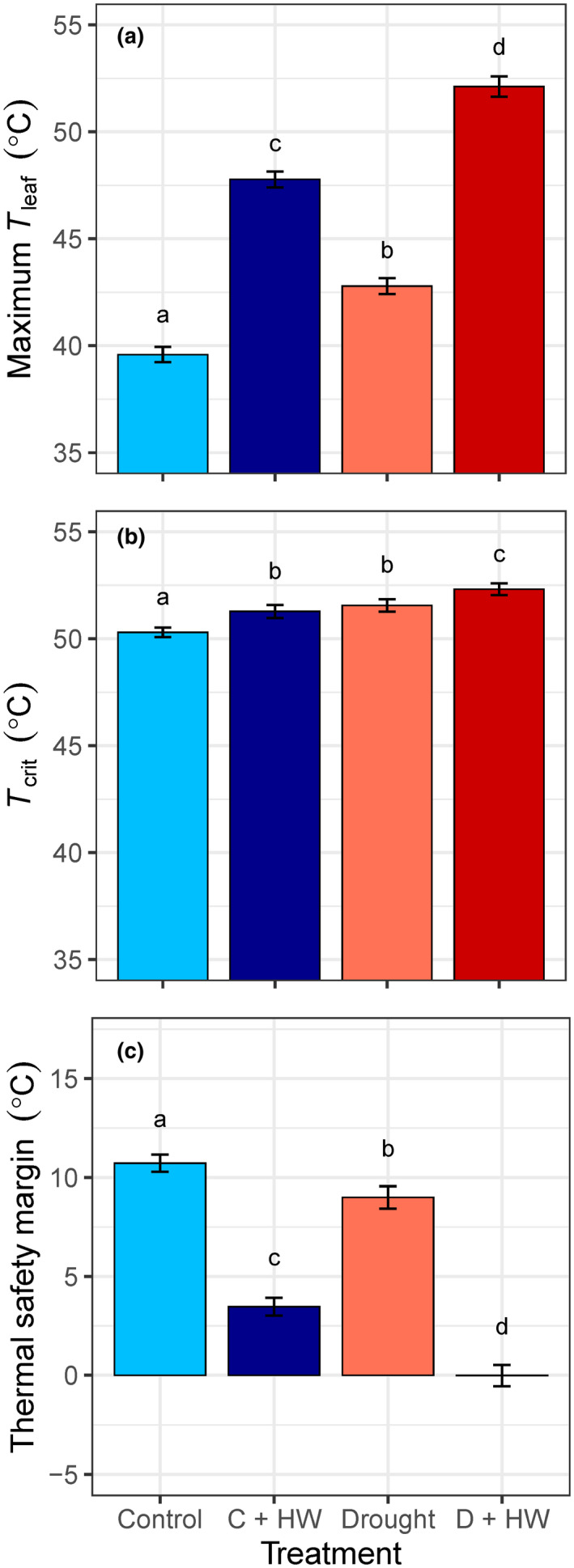
Differences in mean (a) maximum leaf temperature (*T*_leaf_, °C), (b) leaf critical temperature (*T*_crit_, °C), and (c) thermal safety margin (°C) among four experimental treatments (Control, Drought, C + HW: Control + Heatwave, D + HW: Drought + Heatwave) during the fourth (baseline) and fifth (HW) weeks of drought. Values are means (±*SE*) of 73–110 plants per treatment. Means not connected by the same letter are significantly different (Tukey honestly significant difference, *p* < .05)

Our experimental heatwave (+7°C *T*_air_) significantly increased the maximum *T*_leaf_ of well‐watered plants by an average of 8.2°C (*F*_3,351_ = 261.06, *p* < .001; Figure 1a) and *T*_crit_ by 1°C (*F*_3,342_ = 39.22, *p* < .001; Figure 1b) above the control. On average, well‐watered plants maintained positive TSM (i.e., *T*_leaf_ < *T*_crit_) under heatwave temperatures (Figure 1c), with most species avoiding crown dieback (Table S2). The leaves of drought‐stressed plants had a significantly greater risk of overheating (i.e. smaller TSM; *F*_3,315_ = 175.33, *p* < .001), however, due to larger increases in maximum *T*_leaf_ than *T*_crit_ (mean: +3.2 vs. +1.3°C above controls, respectively) under moderate drought compared to well‐watered plants (Figure 1).

**FIGURE 2:**
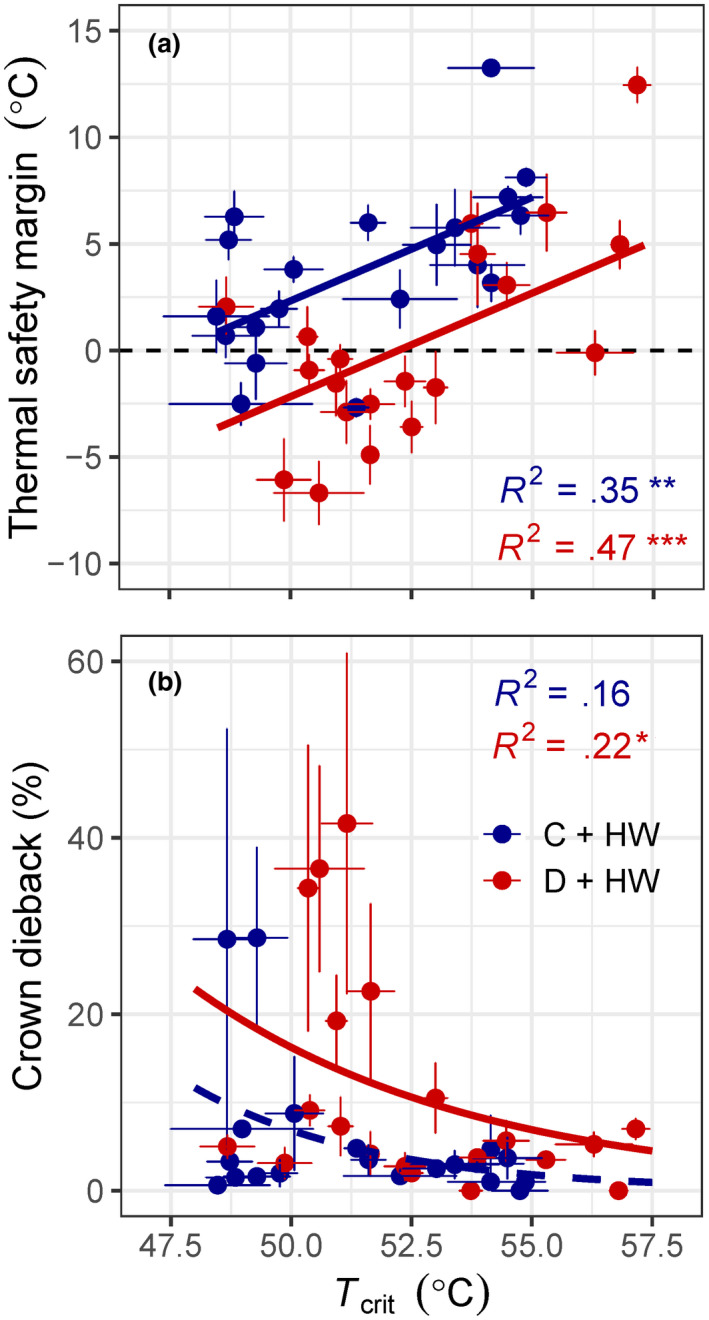
The effect of drought on the relationships between leaf critical temperature (*T*_crit_, °C) and (a) thermal safety margin (°C), (b) crown dieback (%) for 20 broadleaf evergreen tree/shrub species during an experimental heatwave (HW). The control (C + HW) treatment is shown in blue; drought (D + HW) is shown in red. The *T*_crit_ was not significantly correlated to crown dieback for C + HW (*p* = .080). Points are means of 3–9 plants, and error bars indicate *SE*. Asterisks denote significant relationships: *p < .05; **p < .01; ***p < .001

The highest risk of lethal overheating was for drought‐stressed leaves exposed to heatwave temperatures (Figure 1c). Mean *T*_crit_ was highest when drought combined with heat stress, but there was a greater average increase in maximum *T*_leaf_ relative to *T*_crit_ (mean: +12.5 vs. +2°C above controls, respectively; Figure 1a,b). Across all species, the effect of drought significantly decreased leaf TSM ($\chi_{1,39}^{2}$ = 43.94, *p* < .001; Figure 2a) and increased crown dieback ($\chi_{1,39}^{2}$ = 13.67, *p* < .001; Figure 2b) during the heatwave. Six out of 20 species, or about one‐third, experienced >10% crown dieback (Table S3).

### 3.1 Variation in leaf TSM among species

**FIGURE 3:**
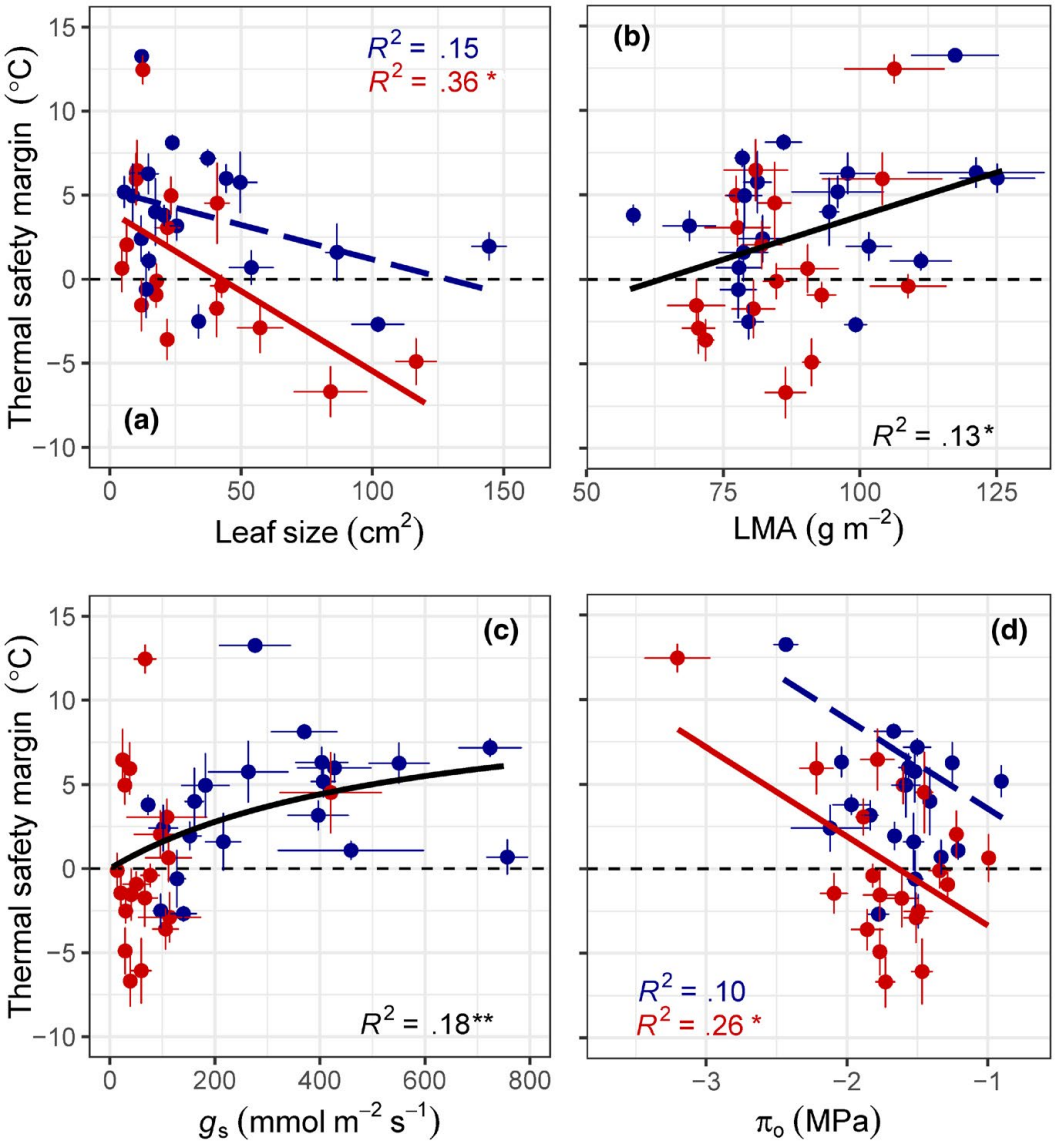
The effect of drought on the relationships between mean (a) leaf size (cm^2^) and (d) leaf osmotic potential (*π*_o_, MPa) and thermal safety margin (TSM, °C) for 20 tree/shrub species during an experimental heatwave (HW). Correlations for leaf size (*p* = .091) and *π*_o_ (*p* = .180) were not significant for C + HW. Species’ mean (b) leaf mass per area (LMA, g m^−2^) and (c) stomatal conductance (*g*_s_, mmol m^−2^ s^−1^) were also significantly correlated with TSM. The C + HW treatment is shown in blue; D + HW is shown in red. Points are means of 3–9 plants, and error bars indicate SE. Asterisks denote significant relationships: *p < .05; **p < .01

We investigated if species’ differences in TSM were related to morphological or physiological leaf traits, including (1) leaf size, (2) LMA, (3) *π*_o_, and (4) *g*_s_. We found that the leaf size was negatively correlated to species’ TSM for droughted plants (*r* ^2^ = .36, *p* = .012) and, to a lesser extent, well‐watered plants (*r* ^2^ = .15, *p* = .091), with the influence of leaf size on TSM depending on drought (*χ* ^2^ = 8.179, *n* = 37, *p* = .004; Figure 3a). Species differences in LMA were positively correlated with TSM (*r* ^2^ = .13, *p* = .031; Figure 3b), such that thick, dense leaves were more likely to maintain positive TSM. Variation in species’ *π*_o_ was negatively correlated to TSM for droughted plants (*r* ^2^ = .26, *p* = .022) but not well‐watered plants (*r* ^2^ = .10, *p* = .180; Figure 3c). Plant species with inherently high *g*_s_ and access to water had larger TSM, relative to species with low *g*_s_ (*r* ^2^ = .18, *p* = .007; Figure 3d). All species with negative TSM had low *g*_s_ (<200 mmol m^−2^ s^−1^), either due to inherently low transpirational capacity or drought‐induced *g*_s_ reductions. We also examined if *T*_leaf_ or *T*_crit_ was more important in explaining variation in species’ TSM and found that the influence of *T*_leaf_ and *T*_crit_ was about equal for droughted plants (Table S4). For well‐watered plants, however, *T*_leaf_ had a greater influence than *T*_crit_ (Table S4).

### 3.2 The effect of heat and drought on stomatal conductance

**FIGURE 4:**
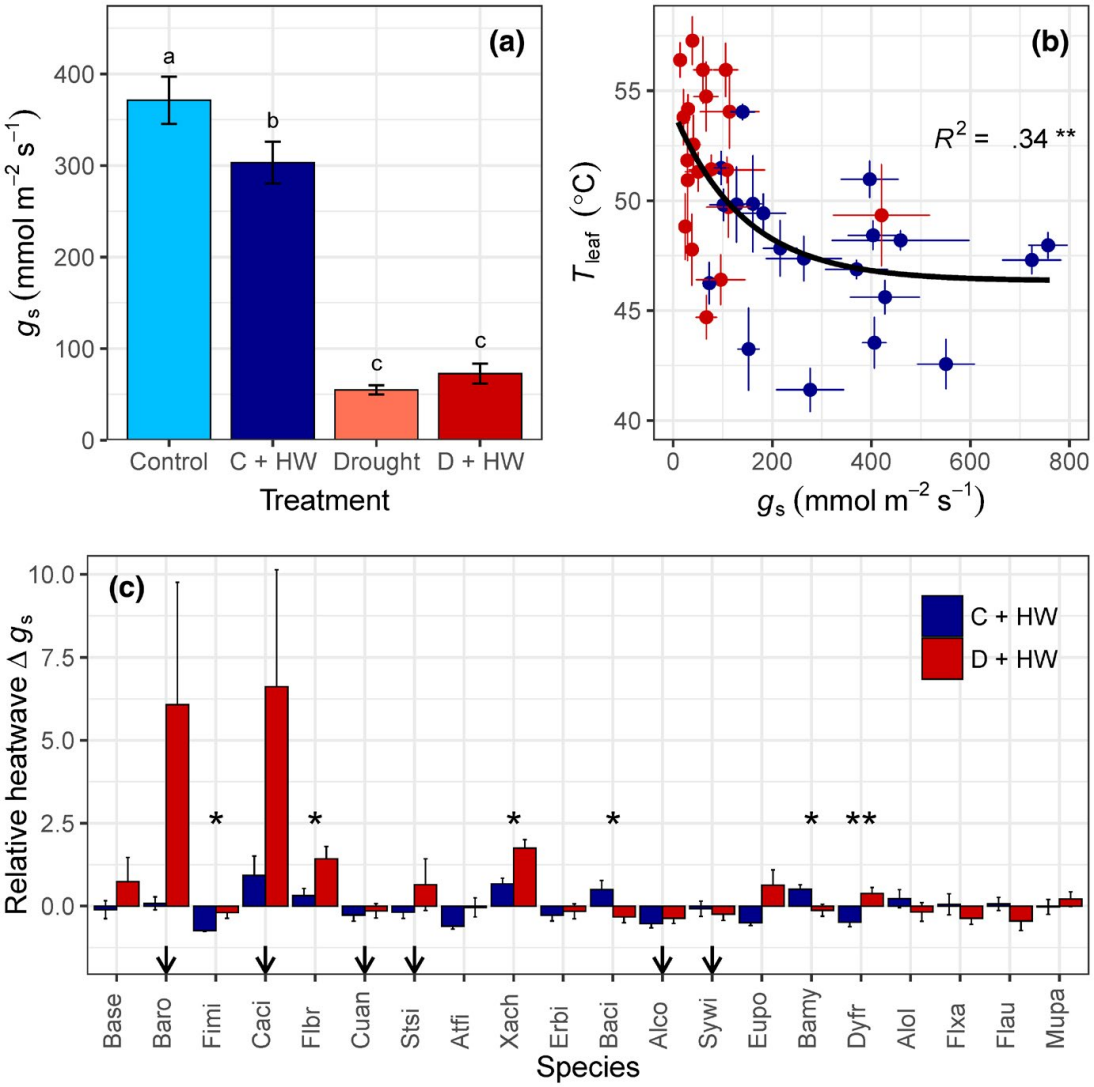
(a) Differences in mean stomatal conductance (*g*_s_, mmol m^−2^ s^−1^) among four experimental treatments (as in Figure 1) during the fourth (baseline) and fifth (heatwave, HW) weeks of drought. Values are means of 91–98 plants per treatment. Means not connected by the same letter are significantly different (Tukey honestly significant difference, *p* < .05). (b) Nonlinear relationship between *g*_s_ and maximum leaf temperature (*T*_leaf_, °C) for 20 tree/shrub species during an experimental heatwave. Points are means of 3–9 plants, and error bars indicate *SE*. (c) The relative change in *g*_s_ from baseline to HW week for 20 tree/shrub species, such that positive numbers represent increased heatwave *g*_s_ (i.e., Δ*g*_s_ of 6 is 6 times higher) and negative numbers represent decreased heatwave *g*_s_. Species are ordered from isohydric to anisohydric and are denoted according to abbreviations in Table S1. Values are means of 3–9 plants, and error bars indicate *SE* (unidirectional *SE* are presented for clarity). Arrows indicate species with crown dieback >10%. Asterisks denote significant differences between relationships and treatments: *p < .05; **p < .01

Stomatal responses to heat depended on plant water availability and differed among species (species × treatment: *F*_3,57_ = 3.86, *p* < .001). On average, we found that well‐watered plants closed stomata and decreased *g*_s_ during the heatwave, whereas droughted plants did not significantly adjust *g*_s_ (*F*_3,375_ = 153.9, *p* < .001; Figure 4a). Stomatal responses varied greatly among species (*F*_3,19_ = 8.89, *p* < .001), however. Well‐watered plants of four species significantly decreased *g*_s_ during the heatwave, relative to baseline, whereas two other species showed the opposite response and significantly increased *g*_s_ (Table S2). Droughted plants of three species significantly increased *g*_s_ under combined drought and heat stress, whereas decreased *g*_s_ was not observed under drought (Table S3). Heatwave‐induced decreases in *g*_s_ had little impact on maximum *T*_leaf_ for species with high *g*_s_, but the relationship between *g*_s_ and *T*_leaf_ was not linear (*r* ^2^ = .34, *p* = .001; Figure 4b) and species with low *g*_s_ experienced larger fluctuations in *T*_leaf_.

### 3.3 Differences between isohydric and anisohydric species

**FIGURE 5:**
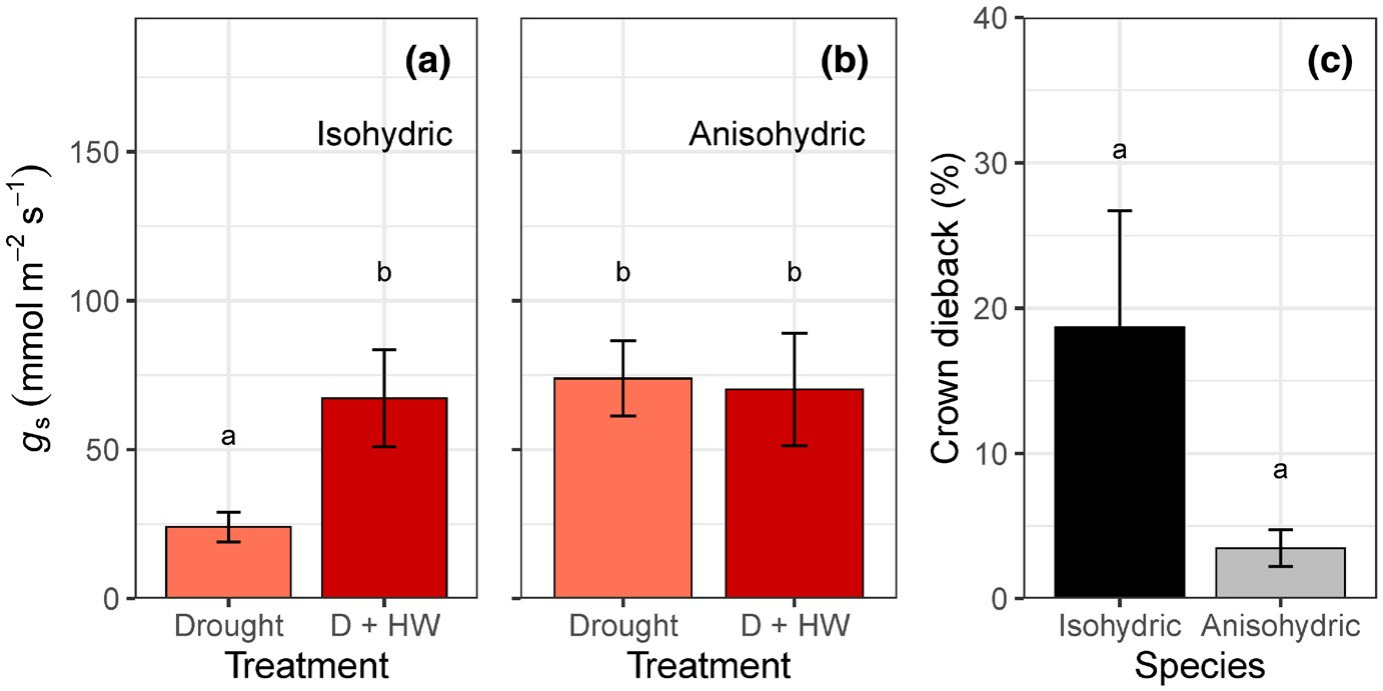
Differences in mean stomatal conductance (*g*_s_, mmol m^−2^ s^−1^) between drought and drought + heatwave (D + HW) experimental treatments for the (a) five most isohydric species (*F*_1,8_ = 5.6, *p* = .046) and (b) five most anisohydric species (*F*_1,8_ = 0.1, *p* = .788) during the fifth (HW) week of drought. (c) Mean crown dieback of D + HW plants was marginally higher in isohydric than anisohydric species (*F*_1,8_ = 4.795, *p* = .060). All values are means of 24 plants per treatment/functional type, and error bars indicate *SE*. Means not connected by the same letter are significantly different (Tukey honestly significant difference, *p* < .05)

We classified all 20 evergreen species along the isohydric to anisohydric continuum by integrating data from *π*_tlp_, wood density, and physiological responses to drought (Table 1). The two extreme functional responses were contrasted by selecting the five most isohydric and five most anisohydric species. Mean *g*_s_ was nearly three times higher for anisohydric than isohydric species under drought (*F*_1,8_ = 12.11, *p* = .008; Figure 5a,b). Isohydric species doubled *g*_s_ under combined heat and drought stress compared to drought alone (*F*_1,8_ = 5.56, *p* = .046; Figure 5a), whereas anisohydric species did not significantly change *g*_s_ (*F*_1,8_ = 0.08, *p* = .788; Figure 5b). Consequently, mean *g*_s_ did not differ between anisohydric and isohydric species under combined heat and drought stress (*F*_1,8_ = 0.1, *p* = .944; Figure 5a,b). Isohydric species were vulnerable to crown dieback during heat and drought stress (mean: 18.7 ± 8%), but anisohydric species avoided crown dieback (mean: 3.5 ± 1%; Figure 5c).

## 4 DISCUSSION

Our study examined the role of thermal stress in contributing to plant mortality during global‐change‐type drought, a topic that has been largely neglected in drought mortality studies to date (Anderegg et al., 2020; Breshears et al., 2021; Trugman et al., 2021). We used a controlled‐environment study to quantify how heatwaves affect plant function by comparing heat responses between well‐watered and drought‐stressed plants. The experimental drought treatment was a gradual, moderate stress event (Marchin et al., 2020) for most species because stomatal closure prevented Ψ_leaf_ from dropping below the turgor loss point (Figure S2c) and thus hydraulic failure was likely avoided (Bartlett et al., 2016). Most plants resumed growth after the heatwave, although plant mortality was observed for two of the 20 broadleaf evergreen species.

It is commonly believed that trees with adequate water supply are generally well adapted to survive transient extreme heat events (Teskey et al., 2015), and our results are consistent. Well‐watered plants significantly increased *T*_crit_ by 1°C during the heatwave (Figure 1b). Acclimation of photosynthesis to higher temperatures can occur within hours of high‐temperature exposure (Havaux, 1993; Hüve et al., 2011; Teskey et al., 2015) via multiple physiological mechanisms, including changes in membrane lipid saturation and accumulation of heat shock proteins, antioxidants, osmolytes, and secondary metabolites (Aspinwall et al., 2019; Wahid et al., 2007). It is worth noting that acclimation to high temperature requires large amounts of energy and is consequently not maintained indefinitely (Wahid et al., 2007). We found that, on average, well‐watered plants maintained positive TSM (i.e. *T*_leaf_ < *T*_crit_) under heatwave temperatures (mean TSM: 3.5 ± 0.5°C; Figure 1c), with most species avoiding crown dieback (Table S2).

The leaves of drought‐stressed plants had a significantly greater risk of overheating, compared to well‐watered plants (i.e., smaller TSM), as a result of larger increases in maximum *T*_leaf_ than *T*_crit_ (Figure 1). Mild droughts can induce increases in thermal tolerance that are typically maintained for at least several weeks after rewatering (Ladjal et al., 2000). The magnitude of drought‐induced increases in *T*_crit_ can be as large as 10°C (Ghouil et al., 2003), but was limited to a maximum of 5°C (above controls) for our study species. Plastic increases in *T*_crit_ are cost‐intensive (Wahid et al., 2007) and depend on species’ acclimation potential in response to dynamic changes in climate (Zhu, Bloomfield, et al., 2018).

The highest risk of lethal overheating was for drought‐stressed leaves exposed to heatwave temperatures (mean TSM: 0 ± 0.5°C; Figure 1c). Mean *T*_crit_ was highest when drought combined with heat stress, but the average increase in maximum *T*_leaf_ exceeded the adjustments in *T*_crit_ (Figure 1a,b). Drought limits the ability of plants to use transpiration to evaporatively cool leaves (Teskey et al., 2015), and cell death can occur within minutes of exposure to lethal high temperatures (Hüve et al., 2011). Our results clearly demonstrate that drought exacerbates thermal damage during heatwaves, confirming our hypothesis that drought‐stressed plants have smaller TSM and greater leaf damage, relative to well‐watered plants (Figure 2).

Species with moderate *T*_crit_ (<52°C) were, unsurprisingly, more at risk of thermal damage (Figure 2a) than species with high *T*_crit_. Two species with very low *T*_crit_ (<50°C) had modest crown dieback (>20%) even when well‐watered (Figure 2b). It is tempting to conclude that species with low *T*_crit_ are more vulnerable to heat stress, but caution is required (Cook et al., 2021; Perez & Feeley, 2020), as *T*_leaf_ can have equal or greater influence than *T*_crit_ on species’ TSM (Table S4). Furthermore, potted plants in glasshouse experiments imperfectly replicate plant responses in the field, where larger rooting volumes may allow better access to soil moisture for improved buffering of *T*_leaf_. Further research is needed to confirm if *T*_crit_ is indeed able to predict species’ vulnerability to heatwaves in the field.

### 4.1 Leaf traits contribute to species differences in TSM

Leaf TSM are a valuable indicator of potential vulnerability to extreme heat, but ideally require the temporal pairing of *T*_crit_ and maximum *T*_leaf_ (Cook et al., 2021) and thus have been collected in relatively few studies to date. Here, we investigated if TSM were related to species differences in leaf morphology and physiology. Across species, we found that TSM were significantly correlated with leaf size, *π*_o_, *g*_s_, and, to a lesser degree, LMA (Figure 3). Leaf size accounted for roughly 35% of the variation in TSM among plant species, at least for droughted plants (Figure 3a). Plant species with large leaves were more likely to experience negative TSM (Figure 3a), which is not particularly surprising, given that large leaves have thick boundary layers that interfere with heat dissipation (Leigh et al., 2017). LMA explained less of the variation among species’ TSM than the other traits examined here; species with thick, dense leaves were more likely to maintain positive TSM (Figure 3b) because thin leaves have low heat buffering capacity (Leigh et al., 2012).

Transpiration is thought to be more effective than leaf physical traits in cooling leaves, at least when water is abundant (Lin et al., 2017). Plant species with inherently high *g*_s_ and access to water had larger TSM, relative to species with low transpirational capacity, whereas all species with negative TSM had low *g*_s_ (<200 mmol m^−2^ s^−1^; Figure 3c). Species with high maximum *g*_s_ typically have higher vein density (McElwain et al., 2016) and small, dense stomata (de Boer et al., 2016) with faster dynamic responses in stomatal aperture (Drake et al., 2013).

Leaf *π*_o_ is important in determining both plant drought tolerance (Bartlett et al., 2012) and plant heat tolerance (Wahid et al., 2007). We found that species with greater accumulation of osmolytes in leaves (i.e., lower *π*_o_) were more likely to maintain positive TSM (Figure 3d). It has been suggested that a common signal triggers both osmotic adjustment and increased *T*_crit_ (Ladjal et al., 2000), perhaps via a single gene (Yang et al., 1996). Taken together, our results suggest that plant heat and drought tolerance are closely related across diverse species and controlled by differences in leaf size, *π*_o_, LMA, and transpirational capacity. Vulnerable species (i.e., small TSM) have low LMA and high maximum *g*_s_, consistent with life history theory. Further study of TSM and associated plant traits holds promise for improving our understanding of which species are most vulnerable during global‐change‐type droughts.

### 4.2 Stomatal responses to extreme heat and drought

Stomata close to prevent excessive water loss under high VPD (Oren et al., 1999), although the controlling mechanism(s) remain poorly understood (Buckley, 2019). We found that, on average, well‐watered plants closed stomata and decreased *g*_s_ during the heatwave (Figure 4a). Stomatal responses to heat depended on species, however, and paradoxically, two species opened stomata and significantly increased *g*_s_ (Table S2). Stomatal closure during heatwaves follows stomatal optimization theory (Cowan & Farquhar, 1977), whereas sacrificing additional water loss under high‐VPD conditions (e.g., heatwaves) contradicts the current stomatal behavior theory (Damour et al., 2010; Lu et al., 2020; Sperry et al., 2017). There has been little evidence for high‐temperature stomatal opening in natural ecosystems, but this may be due to a lack of relevant data under sufficiently high temperatures (>40°C) to date (De Kauwe et al., 2019).

There is a trade‐off between safety and efficiency of stomata, as species with high *g*_s_ have a greater sensitivity for closure during dehydration (Henry et al., 2019). Species with high *g*_s_ were buffered against large fluctuations in maximum *T*_leaf_ (Figure 4b) and maintained larger TSM, relative to species with low *g*_s_ (Figure 3c). Species with low transpirational capacity had smaller TSM (Figure 3c) and risked overheating if stomata closed during a heatwave. Seven species had inherently low *g*_s_ (<200 mmol m^−2^ s^−1^) under well‐watered, control conditions in our experiment, but none of these species reduced *g*_s_ during the heatwave (Table S2). Our results suggest that stomatal closure under extreme heat/VPD is a threshold response only occurring in species with sufficiently high inherent *g*_s_. If so, differences in transpiration rates among species could, at least partially, explain the previously reported contradictory results for stomatal responses to high temperature.

Water‐stressed plants had a fundamentally different stomatal response from the general reduction of *g*_s_ in well‐watered plants (Figure 4a). Droughted plants mirrored the patterns described for species with low transpirational capacity; namely, plants either (1) did not adjust *g*_s_ or (2) significantly increased *g*_s_ under combined drought and heat stress (Table S3). Stomatal opening under high temperatures has been repeatedly observed (Aparecido et al., 2020; Drake et al., 2018; Marchin et al., 2016; Urban et al., 2017) and functions to prevent thermal damage (Schymanski et al., 2013). At low *g*_s_ typical of droughted plants (<150 mmol m^−2^ s^−1^), increases in *g*_s_ had a proportionally larger effect on *T*_leaf_ (Figure 4b) and TSM (Figure 3c). This provides a plausible explanation for why the magnitude of stomatal responses to temperature was generally higher for droughted plants, relative to control plants (Figure 4c). Our results confirmed our second hypothesis, suggesting that droughted plants may be particularly reliant on transpirational leaf cooling and therefore more likely to open stomata during a heatwave.

### 4.3 Stomatal responses to heat differ between isohydric and anisohydric species

To address our third hypothesis, we classified all 20 species along the isohydric to anisohydric continuum by integrating data from *π*_tlp_, wood density (Fu & Meinzer, 2019), and physiological responses to drought (Table 1). The five most anisohydric species tended to have the most negative values of *π*_tlp_ and the highest wood densities, along with smaller reductions in *g*_s_ and larger declines in Ψ_mid_ during experimental drought. Mean *g*_s_ was nearly three times higher for anisohydric than isohydric species under drought (74 vs. 24 mmol m^−2^ s^−1^; Figure 5), as expected. Anisohydric behavior is usually characterized by higher *g*_s_ and transpiration rates under soil water deficit (Tardieu & Simonneau, 1998), but it is unclear how temperature affects typical anisohydric or isohydric stomatal responses.

If the elevated temperature had little influence on stomatal regulation of plants under drought stress, the differences in *g*_s_ between anisohydric and isohydric plants would have been maintained during the heatwave. Instead, we observed that isohydric species doubled *g*_s_ under combined heat and drought stress compared to drought alone (Figure 5a), whereas anisohydric species did not significantly change *g*_s_ (Figure 5b). This disproved our hypothesis, as mean *g*_s_ did not differ between anisohydric and isohydric species under combined heat and drought stress (68 vs. 70 mmol m^−2^ s^−1^; Figure 5). For two isohydric species native to mesic environments, droughted plants increased *g*_s_ by over six times when exposed to high temperatures (*B. robur*, *C. citrinus*; Figure 4c). Interestingly, this stomatal opening was observed at or past their *π*_tlp_ (Figure S4), when stomata of isohydric species are typically closed (Farrell et al., 2017), yet was insufficient at preventing mortality. Other studies have documented stomatal opening under high temperatures for limited species (Aparecido et al., 2020; Drake et al., 2018; Marchin et al., 2016; Urban et al., 2017), but this is the first study to describe a systematic pattern of stomatal opening for isohydric plant species.

Anisohydric species avoided crown dieback without adjusting *g*_s_ during the experimental heatwave (Figure 5), but even low *g*_s_ (e.g., 15% of maximum *g*_s_) is thought to provide effective transpirative cooling to protect against thermal damage (Schymanski et al., 2013). Leaf physical traits may also have contributed to protect these species from crown dieback (Figure 3); small leaf size and high stomatal density allow for greater heat exchange and are better adapted for maintaining temperature in an optimal range (Lin et al., 2017). While we lack data on the potential effect of more extreme temperatures (>42°C), it is logical to speculate that anisohydric plant species may open stomata to benefit from evaporative cooling if leaf temperatures approach their critical threshold. More data are needed to confirm whether the high‐temperature stomatal opening is a universal response for all broadleaf evergreen plants.

## 5 CONCLUSIONS

Previous studies have mainly attributed tree mortality during drought to xylem cavitation and hydraulic failure, without explicitly considering the interactive role of heat stress. We found that some broadleaf evergreen species will paradoxically open stomata during heatwaves, which cools leaves and avoids damaging leaf temperatures but speeds dehydration and risks turgor loss. Actively increasing *g*_s_ and water loss under hot, dry conditions drives plants toward xylem cavitation thresholds more rapidly than has been previously recognized. It is possible that this response is unique to particular species—those with high‐carbon investment in long‐lived leaves (i.e., broadleaf evergreen), isohydric behavior, low transpirational capacity, or native to mesic environments. Further research is needed to determine which species and plant functional types exhibit the same response, and if stomatal responses of potted plants match those of plants in the field. Consideration of leaf traits related to TSM, such as leaf size and stomatal conductance, could help improve our ability to predict the vulnerability of different plant species to future climatic changes. Heat stress can play a critical role in pushing droughted trees closer to mortality thresholds, and as such, should be included as a major mechanism causing tree death during drought.

## CONFLICT OF INTEREST

The authors declare that there is no conflict of interest.

## Supporting information

Supplementary Material

## Data Availability

The raw data supporting the conclusions of this article are publicly available at Figshare (https://doi.org/10.6084/m9.figshare.14883552).
