# Supplementary material for: Extreme heat increases stomatal conductance and drought‐induced mortality risk in vulnerable plant species
Source: Glob Chang Biol. 2021 Nov 20;28(3):1133–46. doi: 10.1111/gcb.15976 (PMC9299030; doi:10.1111/gcb.15976)
Supplement: Supplementary file 1 — Supplementary Material [file GCB-28-1133-s001.docx]

**Supplementary Information**

Extreme heat increases stomatal conductance and drought-induced mortality risk in vulnerable plant species

Renée M. Marchin^1^, Diana Backes^1^, Alessandro Ossola^2^, Michelle R. Leishman^2^, Mark G. Tjoelker^1^, David S. Ellsworth^1^

^1^Hawkesbury Institute for the Environment, Western Sydney University, Penrith, NSW, 2751, Australia

^2^Department of Biological Sciences, Macquarie University, North Ryde, NSW, 2109, Australia

**Corresponding Author:** Renée M. Marchin

Email: [r.prokopavicius@westernsydney.edu.au](mailto:r.prokopavicius@westernsydney.edu.au)

**Contents:**

1. **Tables S1 to S4**
2. **Figures S1 to S4**
3. **Supplementary References**

Table S1. Twenty broadleaf evergreen tree or shrub species selected for this study, including their natural occurrence and climate of origin in Australasia, which is described using the maximum temperature of the warmest month (°C) and precipitation of the driest month (mm)^1^. Climate data are temporal (from 1960‒1990) and spatial (across natural occurrence range) means. Species abbreviation (used in figures) is also provided.

| **Species** | **Species code** | **Natural occurrence** | **Maximum monthly T (°C)** | **Minimum monthly precipitation (mm)** |
| --- | --- | --- | --- | --- |
| *Banksia serrata* L.f. | Base | coastal dry sclerophyll woodland | 25.4 | 57 |
| *Callistemon citrinus* (Curtis) Skeels | Caci | coastal woodland | 25.9 | 55 |
| *Banksia robur* Cav. | Baro | coastal woodland | 26.3 | 58 |
| *Dysoxylum fraserianum* (A.Juss.) Benth. | Dyfr | subtropical rainforest | 27.0 | 47 |
| *Backhousia myrtifolia* Hook. & Harv. | Bamy | subtropical rainforest | 27.2 | 48 |
| *Alectryon coriaceus* (Benth.) Radlk. | Alco | littoral rainforest | 28.2 | 49 |
| *Stenocarpus sinuatus* Endl. | Stsi | subtropical & tropical rainforest | 28.7 | 34 |
| *Flindersia xanthoxyla* (A.Cunn. ex Hook.) Domin | Flxa | dry & subtropical rainforest | 28.7 | 42 |
| *Flindersia brayleyana* F.Muell. | Flbr | tropical rainforest | 29.2 | 46 |
| *Backhousia citriodora* F.Muell. | Baci | subtropical rainforest | 29.4 | 34 |
| *Flindersia australis* R.Br. | Flau | dry & subtropical rainforest | 29.5 | 36 |
| *Syzygium wilsonii* (F.Muell.) B.Hyland | Sywi | tropical rainforest | 29.8 | 47 |
| *Atractocarpus fitzalanii* (F.Muell.) Puttock | Atfi | tropical rainforest | 30.0 | 36 |
| *Cupaniopsis anacardioides* (A.Rich.) Radlk. | Cuan | littoral rainforest | 30.1 | 35 |
| *Ficus microcarpa* (F.M.Bailey) Corner | Fimi | tropical to temperate, >0 °C | 30.6 | 44 |
| *Xanthostemon chrysanthus* (F.Muell.) Benth. | Xach | tropical rainforest | 31.1 | 34 |
| *Murraya paniculata* (L.) Jack | Mupa | tropical to subtropical forest | 31.1 | 30 |
| *Alectryon oleifolius* (Desf.) S.T.Reynolds | Alol | semi-arid woodland | 33.2 | 20 |
| *Eucalyptus populnea* F.Muell. | Eupo | woodland | 33.5 | 26 |
| *Eremophila bignoniiflora* (Benth.) F.Muell. | Erbi | eucalypt woodland | 35.6 | 15 |

^1^ We identified climate of origin using global occurrence records from the *Atlas of Living Australia* (ALA, [www.ala.org.au](http://www.ala.org.au)) and the *Global Biodiversity Information Facility* (GBIF, [www.gbif.org](http://www.gbif.org)) using the *rgbif* and *ALA4R* packages (Chamberlain and Boettiger, 2017) in R Statistical Software [version 3.5.1 (R Core Team, 2018)]. The *CoordinateCleaner* package [version 1.0–7 (Zizka et al., 2019)] was used to remove spatially invalid, duplicate, outdated (pre-1950), outlying (>300 km from other records), and herbaria/capital-city records (<10 km of capital cities). Maximum temperature of the warmest month and precipitation of the driest month were extracted from the *WorldClim Database* [[worldclim.org](https://worldclim.org/), version 1.4 (Hijmans et al., 2005)] for the period 1960‒1990.

Table S2. Stomatal responses of well-watered, control plants during an experimental heatwave, including rates of stomatal conductance (g_s_, mmol m^-2^ s^-1^) under baseline and heatwave conditions, the mean change in g_s_, and direction of significant responses for twenty broadleaf evergreen tree/shrub species. Crown dieback (%) is also given, with values >10% highlighted in bold. The five most isohydric and anisohydric species along the iso/anisohydric continuum are denoted according to the trait ranking described in Table 1.

| **Species** |  | **Baseline g_s_** | **Heatwave g_s_** | **Δ g_s_** | **Response Direction** | **Crown dieback (%)** |
| --- | --- | --- | --- | --- | --- | --- |
|  |  |  |  |  |  |  |
| *Banksia serrata* | Isohydric | 487 ± 47 | 459 ± 139 | ‒ 28 | ‒ | < 5 |
| *Banksia robur* | Isohydric | 754 ± 108 | 757 ± 39 | + 3 | ‒ | **29 ± 24** |
| *Ficus microcarpa* | Isohydric | 619 ± 24 | 161 ± 18 | ‒ 458 | Decrease | < 5 |
| *Callistemon citrinus* | Isohydric | 323 ± 83 | 406 ± 24 | + 83 | ‒ | < 5 |
| *Flindersia brayleyana* | Isohydric | 165 ± 3 | 216 ± 34 | + 51 | ‒ | < 5 |
| *Cupaniopsis anacardioides* |  | 154 ± 31 | 97 ± 14 | ‒ 57 | ‒ | < 10 |
| *Stenocarpus sinuatus* |  | 176 ± 22 | 140 ± 25 | ‒ 36 | ‒ | < 10 |
| *Atractocarpus fitzalanii* |  | 407 ± 51 | 152 ± 22 | ‒ 255 | Decrease | < 5 |
| *Xanthostemon chrysanthus* |  | 425 ± 25 | 724 ± 59 | + 299 | Increase | < 5 |
| *Eremophila bignoniiflora* |  | 857 ± 148 | 551 ± 58 | ‒ 306 | ‒ | < 5 |
| *Backhousia citriodora* |  | 283 ± 67 | 370 ± 63 | + 87 | ‒ | < 5 |
| *Alectryon coriaceus* |  | 551 ± 37 | 264 ± 76 | ‒ 287 | Decrease | < 5 |
| *Syzygium wilsonii* |  | 190 ± 32 | 128 ± 17 | ‒ 62 | ‒ | **29 ± 10** |
| *Eucalyptus populnea* |  | 868 ± 48 | 428 ± 70 | ‒ 440 | Decrease | < 5 |
| *Backhousia myrtifolia* |  | 116 ± 19 | 182 ± 46 | + 66 | Increase | < 5 |
| *Dysoxylum fraserianum* | Anisohydric | 199 ± 24 | 102 ± 28 | ‒ 97 | ‒ | < 5 |
| *Alectryon oleifolius* | Anisohydric | 369 ± 53 | 403 ± 50 | + 34 | ‒ | 0 |
| *Flindersia xanthoxyla* | Anisohydric | 92 ± 34 | 73 ± 11 | ‒ 19 | ‒ | < 10 |
| *Flindersia australis* | Anisohydric | 409 ± 78 | 397 ± 58 | ‒ 12 | ‒ | < 10 |
| *Murraya paniculata* | Anisohydric | 332 ± 79 | 277 ± 68 | ‒ 55 | ‒ | < 5 |

Table S3. Stomatal responses of droughted plants during an experimental heatwave, including rates of stomatal conductance (g_s_, mmol m^-2^ s^-1^) under baseline and heatwave conditions, the mean change in g_s_, and direction of significant responses for twenty broadleaf evergreen tree/shrub species. Crown dieback (%) is also given, with values >10% highlighted in bold. The five most isohydric and anisohydric species along the iso/anisohydric continuum are denoted according to the trait ranking described in Table 1.

| **Species** |  | **Baseline g_s_** | **Heatwave g_s_** | **Δ g_s_** | **Response Direction** | **Crown dieback (%)** |
| --- | --- | --- | --- | --- | --- | --- |
|  |  |  |  |  |  |  |
| *Banksia serrata* | Isohydric | 41 ± 21 | 50 ± 26 | + 9 | ‒ | < 10 |
| *Banksia robur* | Isohydric | 15 ± 1 | 114 ± 60 | + 99 | ‒ | **42 ± 19** |
| *Ficus microcarpa* | Isohydric | 19 ± 5 | 14 ± 4 | ‒ 5 | ‒ | < 10 |
| *Callistemon citrinus* | Isohydric | 20 ± 6 | 112 ± 44 | + 92 | Increase | **34 ± 16** |
| *Flindersia brayleyana* | Isohydric | 24 ± 5 | 60 ± 19 | + 36 | Increase | < 5 |
| *Cupaniopsis anacardioides* |  | 38 ± 10 | 30 ± 11 | ‒ 8 | ‒ | **23 ± 10** |
| *Stenocarpus sinuatus* |  | 36 ± 10 | 39 ± 9 | + 3 | ‒ | **37 ± 12** |
| *Atractocarpus fitzalanii* |  | 36 ± 8 | 29 ± 7 | ‒ 7 | ‒ | < 5 |
| *Xanthostemon chrysanthus* |  | 132 ± 35 | 421 ± 97 | + 289 | Increase | < 5 |
| *Eremophila bignoniiflora* |  | 80 ± 31 | 96 ± 50 | + 16 | ‒ | < 10 |
| *Backhousia citriodora* |  | 45 ± 5 | 28 ± 6 | ‒ 17 | ‒ | 0 |
| *Alectryon coriaceus* |  | 103 ± 22 | 66 ± 25 | ‒ 37 | ‒ | **11 ± 4** |
| *Syzygium wilsonii* |  | 52 ± 14 | 41 ± 15 | ‒ 11 | ‒ | **19 ± 5** |
| *Eucalyptus populnea* |  | 58 ± 9 | 77 ± 18 | + 19 | ‒ | < 10 |
| *Backhousia myrtifolia* |  | 29 ± 6 | 24 ± 5 | ‒ 5 | ‒ | < 5 |
| *Dysoxylum fraserianum* | Anisohydric | 79 ± 21 | 106 ± 25 | + 27 | ‒ | < 5 |
| *Alectryon oleifolius* | Anisohydric | 47 ± 4 | 38 ± 14 | ‒ 9 | ‒ | 0 |
| *Flindersia xanthoxyla* | Anisohydric | 37 ± 6 | 21 ± 5 | ‒ 16 | ‒ | < 5 |
| *Flindersia australis* | Anisohydric | 164 ± 46 | 108 ± 77 | ‒ 56 | ‒ | < 10 |
| *Murraya paniculata* | Anisohydric | 53 ± 5 | 67 ± 21 | + 14 | ‒ | < 10 |

Table S4. Analysis of the contribution of leaf temperature (T_leaf_) versus leaf critical temperature (T_crit_) in explaining variation in species’ mean thermal safety margin (TSM) for both control and droughted plants during an experimental heatwave (C + HW: Control + Heatwave, D + HW: Drought + Heatwave). Both variables were scaled before analysis and then analysed using ordinary least squares regression. The linear relationships between T_crit_ and TSM are shown in Fig. 2A.

| **Treatment** | **T_leaf_** | **T_crit_** | **Residuals** |
| --- | --- | --- | --- |
| C + HW | 57.4% | 34.7% | 7.9% |
| D + HW | 46.5% | 46.7% | 6.8% |


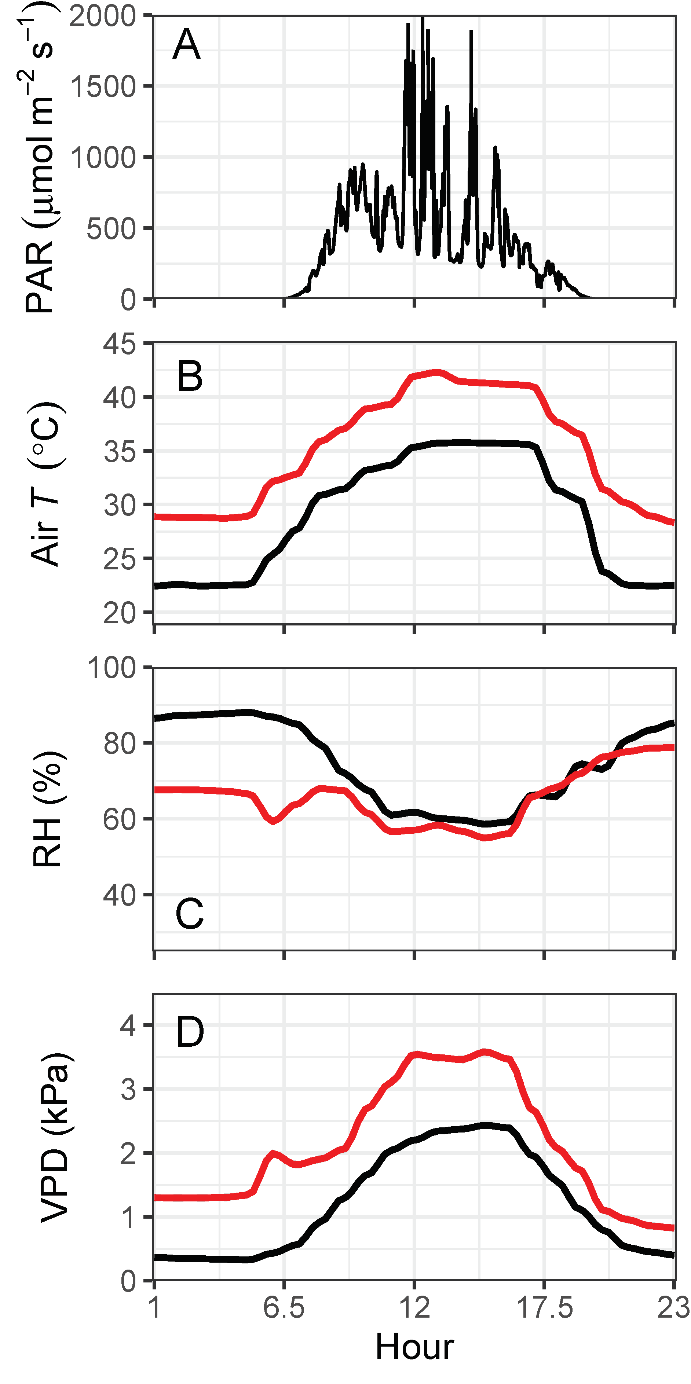


Figure S1. Environmental conditions inside the glasshouse, including (A) photosynthetically active radiation (PAR), (B) mean hourly air temperature, (C) mean hourly relative humidity, and (D) mean hourly vapor pressure deficit during baseline (black line) and heatwave conditions (red line). Data are shown for a representative sunny day (15 January 2019) in the middle of the experiment.


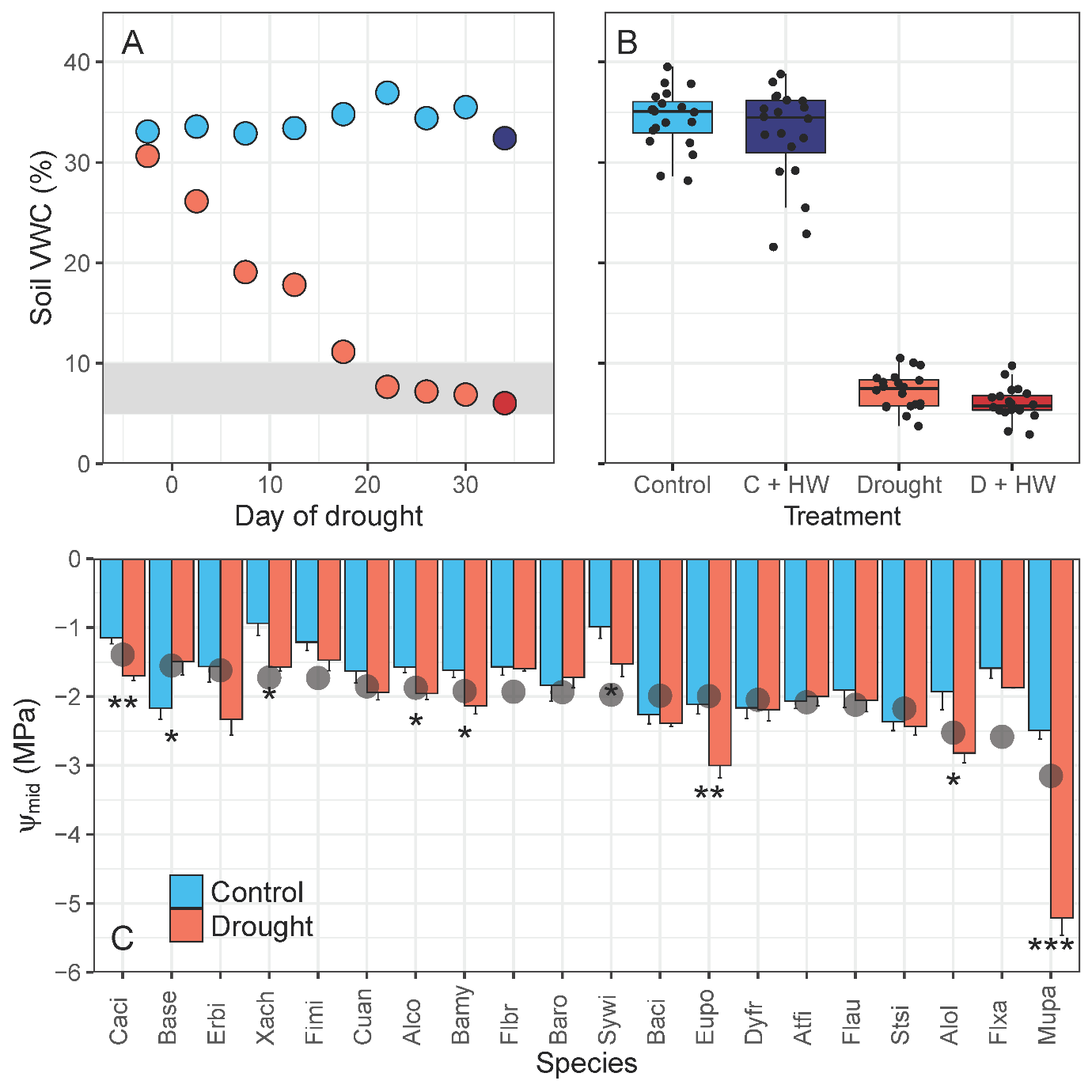


Figure S2. (A) Differences in mean soil volumetric water content (VWC, %) between control (blue) and drought (coral) pots over the five-week experimental drought period; error bars indicate SE but are smaller than the point size. The target drought intensity is shaded gray. Values are means of 46‒105 plants per treatment; sample size varies with day due to binning of data collected in different weeks. (B) Mean soil VWC of four experimental treatments (Control, Drought, C + HW: Control + Heatwave, D + HW: Drought + Heatwave) measured during the fourth (baseline) and fifth (heatwave, HW) weeks of drought. Points are species means of 4–9 plants per treatment; Drought and D + HW are significantly lower than Control and C + HW treatments (*F*_3,438_=1497, *p*<0.001). (C) The effect of drought on midday leaf water potential (Ψ_mid_, MPa) of 20 broadleaf evergreen tree/shrub species during the fourth week of drought. The gray points indicate species mean leaf water potential at turgor loss point (π_tlp_); species are ordered from low to high π_tlp_ and are denoted according to abbreviations in Table S1. Values are means of 4–9 plants, and error bars indicate SE (unidirectional SE are presented for clarity). Asterisks denote significant differences between treatments: * *p*<0.05; ** *p*<0.01; *** *p*<0.001.


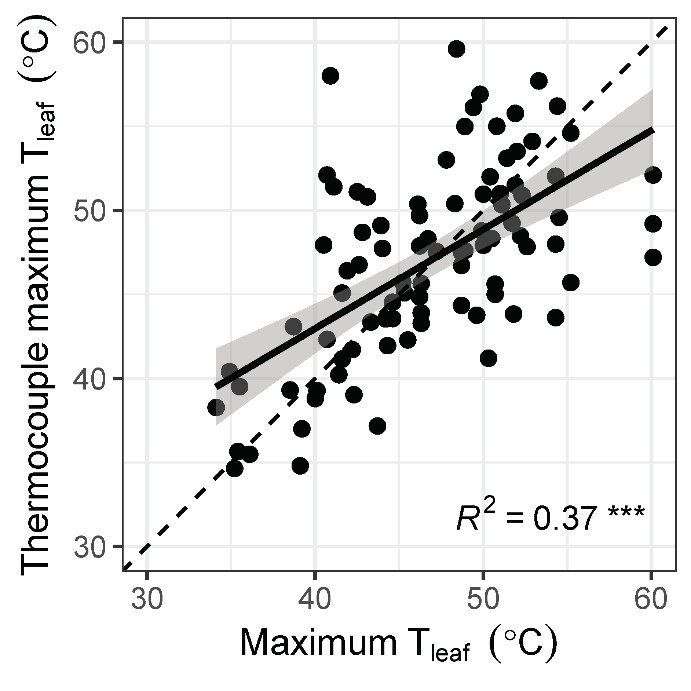


Figure S3. Comparison of maximum leaf temperature (T_leaf_) measured either as point measurements using an infrared thermometer (x-axis) or as continuous measurements using leaf thermocouples (y-axis) for plants from a subset of nine plant species for which continuous measurements were available. The relationship was analyzed using standardized major axis (SMA) regression, as it is not possible to functionally assign either parameter as a dependent variable. The two independent methods were significantly correlated (*r*^2^=0.37, *p*<0.001) and surrounded the 1:1 line (dashed line), so all measurements were pooled to determine maximum T_leaf_ for each plant.


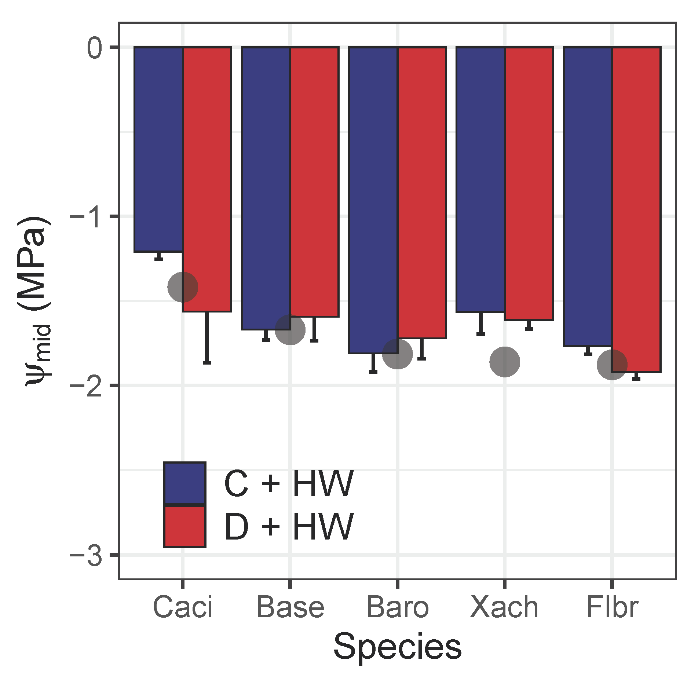


Figure S4. The effect of drought on midday leaf water potential (Ψ_mid_, MPa) for five broadleaf evergreen tree/shrub species with large relative increases in stomatal conductance under the combination of heat and drought stress (see Fig. 3C). The gray points indicate species’ mean leaf water potential at turgor loss point (π_tlp_); species are ordered from low to high π_tlp_ and are denoted according to abbreviations in Table S1. Values are means of 4–5 plants, and error bars indicate SE (unidirectional SE are presented for clarity).

**Supplementary References**

Chamberlain, S.A., and Boettiger, C. (2017). R Python, and ruby clients for GBIF species occurrence data. *PeerJ Preprints* 5. doi: 10.7287/peerj.preprints.3304v1.

Hijmans, R.J., Cameron, S.E., Parra, J.L., Jones, P.G., and Jarvis, A. (2005). Very high resolution interpolated climate surfaces for global land areas. *International Journal of Climatology* 25(15)**,** 1965-1978. doi: 10.1002/joc.1276.

R Core Team (2018). R: A language and environment for statistical computing (Version 3.5.1, R Foundation for Statistical Computing, Vienna, Austria).

Zizka, A., Silvestro, D., Andermann, T., Azevedo, J., Ritter, C.D., Edler, D., et al. (2019). CoordinateCleaner: Standardized cleaning of occurrence records from biological collection databases. *Methods in Ecology and Evolution* 10(5)**,** 744-751. doi: 10.1111/2041-210x.13152.
